# Attenuation of *Listeria monocytogenes* Virulence by *Cannabis sativa* L. Essential Oil

**DOI:** 10.3389/fcimb.2018.00293

**Published:** 2018-08-22

**Authors:** Emanuela Marini, Gloria Magi, Gianna Ferretti, Tiziana Bacchetti, Angelica Giuliani, Armanda Pugnaloni, Maria Rita Rippo, Bruna Facinelli

**Affiliations:** ^1^Unit of Microbiology, Department of Biomedical Sciences and Public Health, Polytechnic University of Marche, Ancona, Italy; ^2^Department of Clinical Sciences, Polytechnic University of Marche, Ancona, Italy; ^3^Department of Life and Environmental Sciences, Polytechnic University of Marche, Ancona, Italy; ^4^Division of Pathology, Department of Clinical and Molecular Sciences, Polytechnic University of Marche, Ancona, Italy

**Keywords:** *Cannabis sativa*, essential oil, sublethal concentrations, *Listeria monocytogenes*, virulence, motility, cell invasion, biofilm

## Abstract

Anti-virulence strategies are being explored as a novel approach to combat pathogens. Such strategies include inhibition of surface adhesion, tissue invasion, toxin production, and/or interference with the gene regulation of other virulence traits. *Listeria monocytogenes*, the causative agent of listeriosis, is a facultative intracellular food pathogen characterized by a wide distribution in the environment. Its ability to persist within biofilms and to develop resistance to sanitizers is the cause of significant problems in food processing plants and of steep costs for the food industry. In humans, the treatment of listeriosis is hampered by the intracellular location of listeriae and the poor intracellular penetration of some antibiotics. Eleven *L. monocytogenes* isolates from patients who were diagnosed with invasive listeriosis in Italy in 2014–2016 were studied. This *in vitro* and *in vivo* study explored the antibacterial and anti-virulence properties of a steam-distilled essential oil of *Cannabis sativa* L., which is being intensively investigated for its high content in powerful bioactive phytochemicals. Susceptibility experiments demonstrated a moderate bactericidal activity of the essential oil (Minimum Bactericidal Concentration > 2048 μg/mL). Assessment of the effects of sublethal concentrations of the essential oil on *L. monocytogenes* virulence traits demonstrated a significant action on motility. Listeriae were non-motile after exposure to the essential oil. Light and scanning electron microscopy documented aggregates of listeriae with the flagella trapped inside the cluster. Real-time RT-PCR experiments showed downregulation of flagellar motility genes and of the regulatory gene *prfA*. The ability to form biofilm and to invade Caco-2 cells was also significantly reduced. *Galleria mellonella* larvae infected with *L. monocytogenes* grown in presence of sublethal concentrations of the essential oil showed much higher survival rates compared with controls, suggesting that the extract inhibited tissue invasion. Food contamination with *L. monocytogenes* is a major concern for the food industry, particularly for plants making ready-to-eat and processed food. The present work provides a baseline in the study of the anti-virulence properties of the *C. sativa* essential oil against *L. monocytogenes*. Further studies are needed to understand if it could be used as an alternative agent for the control of *L. monocytogenes* in food processing plants.

## Introduction

In recent years, the use of plant products as alternative/adjunct antimicrobial agents to control pathogenic microorganisms has been attracting mounting interest. Anti-virulence strategies are being explored as a novel approach to combat bacterial pathogens; such approaches include inhibition of surface adhesion, tissue invasion, toxin production, and/or interference with the gene regulation of other virulence traits (Rasko and Sperandio, 2010). A major group of plant antimicrobial compounds is represented by essential oils (EO), complex mixtures of volatile secondary metabolites belonging to different chemical families including terpenes, alcohols, ethers, aldehydes, and phenols (Cannas, 2016). The antimicrobial activity of EO and their components has been known for millennia. More recently, synergy between EO components and antibiotics has been demonstrated against antibiotic-resistant pathogens (Langeveld et al., 2014). In contrast, the knowledge of the effects of EO on bacterial virulence is still very limited (Nazzaro et al., 2013; Silva et al., 2016).

*Cannabis sativa* L. (Cannabaceae), an annual species that has spread from its native range in central Asia to Europe and Africa, is one of the earliest domestic plants in the history of mankind and has been grown and selected for thousands of years for a multiplicity of purposes, like a source of food, fuel, paper, and building material, as a textile fiber, and as a remedy in folk medicine (Andre et al., 2016). The plant is a source of several bioactive compounds including psychoactive substances such as cannabinoids (the most important being Δ9-tetrahydrocannabinol, Δ9-THC), terpenoids, flavonoids, and polyunsaturated fatty acids. In the late 1930s, the psychotropic effects due to Δ9-THC led to a ban on its cultivation worldwide. In recent years, selection of some genotypes containing low Δ9-THC concentrations (≤0.2% w/v) has led to a lifting of the ban; the plant can now be grown legally (Holler et al., 2008) and used for research purposes.

*Listeria monocytogenes*, a Gram-positive, facultative intracellular pathogen with a wide environmental distribution, is the causative agent of human and animal listeriosis (Freitag et al., 2009). The disease is the most lethal zoonosis in the EU (fatality rate, 20%), affecting particularly immunocompromised and elderly individuals (EFSA and ECDC, 2016). Infection of the human host is commonly via the oral route, through ingestion of contaminated food, although transplacental transmission during gestation also occurs. Severe *L. monocytogenes* infection include septicaemia, meningitis, endocarditis, and spontaneous abortion (McDougal and Sauer, 2018). Infection requires bacterial internalization into host cells, intracellular survival, and spread into neighboring cells, which enable bacterial diffusion from the primary site of infection, usually the bowel, to the liver, the spleen, and on to peripheral blood and eventually the brain. A limited subset of serotypes (i.e., 1/2a, 1/2b, and 4b) is responsible for the bulk of clinical cases worldwide (Kathariou, 2002); the serotype most frequently associated with listeriosis outbreaks, particularly in Europe and North America, is 1/2a (Lomonaco et al., 2015). *L. monocytogenes* is intrinsically resistant to broad-spectrum cephalosporin antibiotics (Krawczyk-Balska and Markiewicz, 2016) and shows acquired resistance to other drugs. Treatment with aminopenicillin or benzylpenicillin, alone or combined with an aminoglycoside, is currently the gold standard antibiotic therapy for *L. monocytogenes* infections (Thønnings et al., 2016). However, listeriae are difficult to eradicate, due to their intracellular location and the poor intracellular penetration of these drugs. Their ability to persist within biofilms and to develop resistance to sanitizers is the cause of significant problems in food processing plants and of steep costs for the food industry (Colagiorgi et al., 2017; Boqvist et al., 2018).

The virulence of *L. monocytogenes* relies on highly effective mechanisms of invasion and spread, which ensure its intracellular lifecycle (Vázquez-Boland et al., 2001; Freitag et al., 2009; Radoshevich and Cossart, 2018). Host cell invasion is either via phagocytosis or receptor-mediated endocytosis, facilitated by the bacterial surface proteins internalins InlA (*inlA*) and InlB (*inlB*) (Chen et al., 2017). Recently, Drolia et al. have demonstrated that *Listeria* adhesion protein (LAP) induces intestinal epithelial barrier dysfunction contributing to bacterial translocation from the intestinal lumen, across the gut epithelium (Drolia et al., 2018). *L. monocytogenes* is initially trapped in a phagocytic vacuole and subsequently secretes the cholesterol-dependent pore-forming toxin listeriolysin O (LLO; *hly*) and two phospholipases C (PlcA and PlcB), resulting in vacuole rupture and escape of bacteria into cytosol (Chen et al., 2017). In this phase, an actin assembly-inducing protein (ActA; *actA*) promotes actin-based motility and cell-to-cell spread (Chen et al., 2017). These processes are closely regulated, mainly by the transcriptional regulator PrfA (*prfA*), which is activated by host infection (de las Heras et al., 2011). Recent progress highlights that *L*. *monocytogenes*, which has long been a model for cytosolic pathogens, is also capable of residing in vacuoles, in a slow/non-growing state (Bierne et al., 2018).

Flagellum-based motility and biofilm formation have also been implicated in virulence (Josenhans and Suerbaum, 2002; Duan et al., 2013). The biosynthesis of flagella is temperature-dependent, since most *L. monocytogenes* strains produce flagella and are motile only at temperatures of 30°C and less (Peel et al., 1988). Flagellum-mediated motility is critical both for initial surface attachment and subsequent biofilm formation (Lemon et al., 2007).

The purpose of this study was to investigate the *in vitro* and *in vivo* antibacterial and anti-virulence properties of an EO extracted from a legal *C. sativa* L. variety against *L. monocytogenes* isolates collected from patients diagnosed with invasive listeriosis in central Italy in 2014-2016.

## Materials and methods

### Strains and growth media

A total number of 11 *L. monocytogenes* isolates collected from the blood or cerebrospinal fluid (CSF) of patients diagnosed with invasive listeriosis in Ancona province (Italy) from 2014 to 2016 were investigated in the study (Table 1). The 2015 and 2016 isolates had already been characterized in terms of antibiotic resistance phenotype and molecular typing/genetic relatedness (Marini et al., 2016).

**Table 1:** *L. monocytogenes* strains used in the study and their characteristics.

**Strain #**	**Date of isolation**	**Source**	**Serotype**	**MBC (**μ**g/mL)**			**Biofilm producer**
				**EO**	**α-pinene**	**Myrcene**	
60551	2014	Blood	4b	>2048	1024	2048	Weak
66785	“	“	1/2a	“	>2048	“	Strong
75227	“	“	1/2b	“	1024	“	Moderate
82468	“	“	1/2a	“	2048	“	Strong
97885	“	“	4b	“	>2048	“	Moderate
02470	2015	“	1/2a	“	“	>2048	Strong
09707	“	“	1/2c	“	“	2048	Moderate
56053	“	“	1/2a	“	1024	“	Moderate
31829	“	“	“	“	2048	“	Moderate
77660	2016	CSF	4b	“	1024	“	Moderate
80466	“	Blood	1/2a	“	2048	“	Moderate

*CSF, cerebrospinal fluid; MBC, minimum bactericidal concentration*.

Blood agar base (BAB) supplemented with 5% sheep blood, Müller-Hinton agar (MHA) supplemented with 5% sheep blood, Müller-Hinton cation-adjusted broth (CAMHB) supplemented with 3% laked sheep blood, brain heart infusion (BHI) agar and broth, Tryptone Soya Broth (TSB), and Luria Bertani (LB) agar and broth (all from Oxoid, Basingstoke, UK) were used in the study. Isolates were maintained in glycerol at −70°C and subcultured twice on BAB before testing.

### Extraction and characterization of *C. sativa* L. essential oil

EO was extracted from the French monoecious variety Futura 75, which is grown in the countryside around Ancona. Fresh inflorescences and leaves were steam-distilled by the Associazione Produttori Piante Officinali (APPO Marche, Ancona, Italy). Gas chromatography/mass spectrometry (GC-MS) identification of EO components was performed by Agenzia Servizi Settore Agroalimentare delle Marche (ASSAM, Ancona, Italy).

### Susceptibility tests

The minimum inhibitory concentration (MIC), i.e., the lowest EO concentration inhibiting visible bacterial growth after incubation, was determined in blood-supplemented CAMHB by the microdilution method according to Clinical and Laboratory Standards Institute (CLSI) guidelines (Clinical Laboratory Standards Institute, 2017). The minimum bactericidal concentration (MBC), i.e., the lowest EO concentration killing 99.9% of the inoculum, was determined by plating 10 μL of each microdilution on blood-supplemented MHA followed by overnight incubation at 37°C. All experiments were performed in triplicate.

### Motility assays

Motility of *L. monocytogenes* in presence of the *C. sativa* EO was assessed by the “umbrella test” in semi-solid agar stabbed with the strains and incubated overnight at 30°C. Detection of an umbrella-like growth about 0.5 cm below the agar surface indicated a positive test. Motility was also assessed by measuring the diameter of the growth zone with respect to control bacteria on 0.3% soft agar plates inoculated with 10 μL of a broth culture and incubated overnight at 30°C.

### Flagella stain

*L. monocytogenes* incubated at 30°C in presence of the *C. sativa* EO were examined by light microscopy (LM). Flagella were visualized using BD Flagella Stain Droppers (Becton Dickinson, Milano, Italy), a procedure that demonstrates bacterial flagella and their arrangement on the cell.

### Scanning electron microscopy examination

For morphological studies, *L. monocytogenes* cells grown on BAB agar at 30°C with and without the *C. sativa* EO were fixed overnight in 2.5% glutaraldehyde in 0.1 M sodium cacodylate buffer (pH 7.4) at 4°C and washed with the same buffer supplemented with 7% sucrose. Samples were left to adhere on glass slides covered with 0.01% poly-L-lysine solution (Sigma Aldrich, Milano, Italy), postfixed in 1% OsO_4_ in 0.1 M sodium cacodylate buffer for 2 h at 4°C, washed with the same buffer, dehydrated with EtOH, and critical point dried. Samples were mounted on aluminum stubs by graphite glue (Sigma Aldrich) and coated with a thin (20 Å) gold film using an EMITECH K550 (Ashford, England) sputtering device. Scanning electron microscopy (SEM) observations were performed with a Zeiss Supra 40 apparatus.

### RNA extraction and real-time quantitative reverse transcription PCR (RT-qPCR) on *prfA, flaA, motA*, and *motB*

The effect of the EO on the expression of *L. monocytogenes* virulence genes was investigated using real time RT-PCR. Strains were grown in presence of EO at 30°C in TSB, and total RNA was extracted using GenElute total RNA Purification kit (Sigma Aldrich). cDNA synthesis was performed using the Quantitect Reverse Transcription kit (Qiagen, Hilden, Germany) and synthesized cDNA was used as the RT-qPCR template. The amplification product was detected using SYBR Green reagents (SYBR Green JumpStar Taq ReadyMix, Sigma Aldrich) by Rotor-Gene (Qiagen). The primers for each gene are reported in Table 2. Data were normalized to the endogenous control (16S rRNA) and the level of candidate gene expression between treated and untreated samples was compared to study relative gene expression and the effect of EO on each gene.

**Table 2:** List of primers used in the study.

**Gene**	**Primer**	**Sequence (5^′^- 3^′^)**	**Product size**
*16S*	rDNA 16s-F	AGGTGGGGATGACGTCAAAT	138 bp
	rDNA 16s-R	GCAGCCTACAATCCGAACTG	
*prfA*	prfA-RT-F	GACCGCAAATAGAGCCAAGC	122 bp
	prfA-RT-R	ACTGAGCAAGAATCTTACGCAC	
*flaA*	flaA-RT-F	GGCTGCTGAAATGTCCGAAA	90 bp
	flaA-RT-R	ATTTGCGGTGTTTGGTTTG	
*motA*	motA-RT-F	GGTTACGTACTTTGGACGCC	137 bp
	motA-RT-R	AAACGTTCTTCCACAACCCG	
*motB*	motB-RT-F	CGTTCTGTTTGCCTCCAGTT	103 bp
	motB-RT-R	ATATGCTTGATTGCCTGCCG	

### Biofilm formation assays

Biofilm formation was evaluated as previously described (Marini et al., 2015). Briefly, bacteria were grown overnight in TSB containing 1% glucose at 30°C. Overnight bacterial suspensions were prepared to yield final inocula of ~1 × 10^8^ colony-forming units (CFU)/mL; then 200 μL aliquots of the bacterial suspension were inoculated into 96-well microtitre plates at least in triplicate. After 24-h incubation, wells were washed 3 times in phosphate-buffered saline (PBS), dried for 1 h at 60°C, and stained with Hucker's crystal violet. After three washes in sterile water, wells were inoculated with 100 μL of 95% EtOH and shaken for 10 min. Biofilm formation was quantified by measuring absorbance at 690 nm with a Multiscan Ascent apparatus (Thermo Scientific, Waltham, MA, USA). The optical density (OD) cut-off (ODc) was defined as 3 standard deviations above the mean OD of the negative control, represented by non-inoculated wells containing TSB (Stepanovic et al., 2000). Strains were classified as non-producers (OD ≤ ODc), weak producers (ODc < OD ≤ 2 × ODc), moderate producers (2 × ODc < OD ≤ 4 × ODc), or strong producers (OD > 4 × ODc). The biofilm-forming strain *S. epidermidis* ATCC 35984 was used as a positive control (Christensen et al., 1985). In some assays, biofilm formation was evaluated in presence of the *C. sativa* EO. Briefly, overnight bacterial suspensions were prepared to yield final inocula of ~2 × 10^8^ CFU/mL. Then, 100 μL of bacterial culture and 100 μL of different EO concentrations were added to each well of a 96-well microplate. Wells containing 100 μL of the bacterial suspension and 100 μL TSB without EO were the positive controls. After incubation, biofilm formation was evaluated as described above. All experiments were performed in triplicate.

### Caco-2 invasion assays

The human colon carcinoma Caco-2 cell line (ATCC HTB37) (Rousset, 1986) was used in cell invasion experiments. Cells were routinely cultured in Modified Eagle Medium (MEM; Gibco, New York, NY, USA) supplemented with 1% (v/v) L-glutamine, 1% (v/v) non-essential amino acids and 10% (v/v) fetal calf serum (both from Gibco) in 50 mL (25 cm^2^) plastic tissue culture flasks (Corning Costar, Milano, Italy) at 37°C in an atmosphere containing 5% CO_2_. Monolayers were trypsinized and adjusted to a concentration of 2.5 × 10^5^ cells/mL in culture medium; 1 mL cell suspension was dispensed into each 22-mm well of a 12-well tissue culture plate and incubated 4 days to obtain confluent monolayers (Facinelli et al., 1998).

Invasion experiments were performed using *L. monocytogenes* strains grown in presence of the *C. sativa* EO. Briefly, after overnight growth in BHI broth supplemented with EO, listeriae were resuspended in PBS to OD_550_ 0.6 ± 0.02. Then, 1 mL of the suspension, suitably diluted in MEM, was added to each well to obtain a multiplicity of infection of about 30 bacteria/cell. Penetration was allowed to occur for 1 h at 37°C in 5% CO_2_. Then monolayers were washed 3 times and covered with 2 mL MEM containing a bactericidal concentration of gentamicin (10 μg/mL), to kill extracellular bacteria. After 2 h at 37°C in 5% CO_2_ air, cells were washed 3 times and lysed in 1 mL Triton X-100 (0.1% in cold sterile water). The CFU of viable bacteria were counted by plating suitable dilutions of the lysates on BHI agar and incubating them for 36-48 h at 37°C. Data are expressed as a percentage of initial inoculum of viable bacteria. Experiments were carried out in triplicate.

### Cytopathogenic effects on Caco-2 monolayers

The ability of listeriae to cause cytopathogenic effects (CPE) in Caco-2 monolayers was evaluated by the trypan blue exclusion assay (Gibco), which measures cell viability (Facinelli et al., 1998). Briefly, Caco-2 monolayers grown in slide flasks (Corning Costar) were infected with bacterial strains grown with and without the *C. sativa* EO, as described above. Control monolayers received MEM without bacteria. At the end of the infection period (1 h), the supernatant was removed and monolayers were washed and covered with MEM supplemented with gentamicin (10 μg/mL). After overnight incubation at 37°C in 5% CO_2_, 1 mL of a 0.4% trypan blue solution was added to the culture medium and cells were kept at room temperature for 30 min. Finally, the culture medium with the dye was removed and monolayers were examined with a Leica DMRB microscope (Leica Microsystems, Wetzlar, Germany) using a 10 × objective.

### *Galleria mellonella* survival assays

Final instar larvae (~300 mg) of the greater wax moth *G. mellonella* were purchased from a local vendor, stored in the dark at 15°C, and used within 7 days. Larvae were rinsed with 70% EtOH before injection using a standard protocol (Mukherjee et al., 2010). Ten larvae were randomly allocated to different treatment groups: positive control (larvae inoculated with *L. monocytogenes*); test group (larvae injected with *L. monocytogenes* grown in presence of the *C. sativa* EO); negative control (larvae that were not injected); PBS control (larvae injected with PBS). Before infection, bacteria were washed and resuspended in PBS at the appropriate cell density (5 × 10^7^ CFU/mL); then, 50 μL of the inoculum was injected into the larval haemocoel via the left proleg using a microsyringe (Mukherjee et al., 2010). After injection, larvae were incubated at 30°C in sterile Petri plates. The number of dead larvae was scored at 24 h intervals for 6 days. Larvae were considered dead when they turned black and failed to react to touch. Three separate experiments were conducted. *G. mellonella* survival data were plotted using the Kaplan-Meier method.

### Statistics

Data are mean ± standard deviation (SD). Differences between groups were assessed using GraphPad Prism, version 5 (GraphPad Software Inc., San Diego, CA, USA) by a paired Student's *t*-test (biofilm, cell invasion and real time RT-PCR experiments) and the log-rank (Mantel-Cox) test (*G. mellonella* survival assays). *P* ≤ 0.05 indicated statistical significance.

## Results

### GC-MS analysis of the *C. sativa* EO

GC-MS analysis of the *C. sativa* EO, obtained from the Futura 75 genotype by steam distillation, identified 35 compounds, which accounted for 95% of the whole GC profile (Table 3). Most were sesquiterpenes found at very low concentrations, except for β-caryophyllene (15.31%) and α-humulene (3.68%). Myrcene (17.17%), α-pinene (19.20%), and β-pinene (4.34%) were the main monoterpenes.

**Table 3:** GC-MS analysis of the *C. sativa* EO.

**Components**	**%**
	
Tricyclene	0.63
α-thujene	0.09
α**-pinene**	**19.20**
Camphene	0.65
β**-pinene**	**4.34**
**Myrcene**	**17.17**
δ-3-carene	0.60
α-phellandrene	0.15
Limonene	1.81
β-phellandrene	0.86
1,8-cineole	1.06
p-cymene	1.52
Trans-β-ocimene	2.28
γ-terpinene	0.12
**Terpinolene**	**4.50**
Neo-allo-ocimene	0.14
**Camphor**	**5.78**
Borneol	1.15
Terpinen-4-ol	0.28
α-terpinene	0.46
Cuminol	0.64
Verbenone	0.95
Bornyl acetate	0.56
Ethyl hexanoate	0.08
Iso-caryophyllene	0.94
β**-caryophyllene**	**15.31**
Allo-aromadendrene	0.11
Trans-β-farnesene	1.11
α**-humulene**	**3.68**
γ-selinene	0.28
β-selinene	1.35
α-selinene	1.08
Valencene	1.22
**Caryophyllene-oxide**	**3.27**
α-bisabolol	0.61
	

*Components present at greater than 3% are indicated by bold font*.

### Susceptibility of *L. monocytogenes* strains to the *C. sativa* EO and its main components

The antimicrobial activity (MIC and MBC) of the total EO, α-pinene, and myrcene was determined against all tested strains. Only the MBC values are reported, because the MIC values were difficult to interpret due to turbidity (Table 1). The MBC of the whole EO and of myrcene were ≥ 2048 μg/mL, whereas those of α-pinene ranged from 1024 to > 2048 μg/mL, suggesting that both components contribute to the bactericidal activity of the extract against the *L. monocytogenes* strains.

### Reduced flagellar motility in presence of the *C. sativa* EO

**Figure 1:**
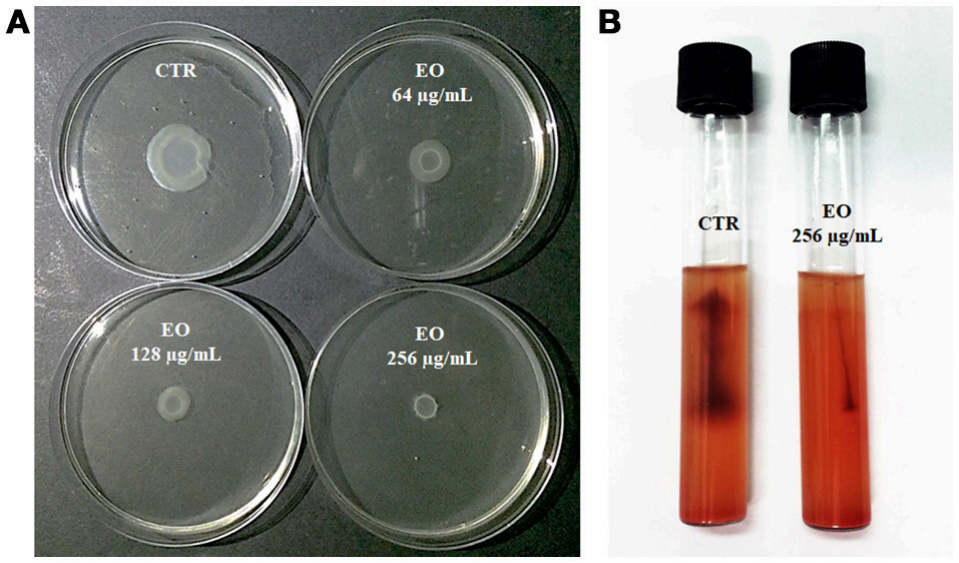
Motility of *L. monocytogenes* strain #80466 in presence of the *C. sativa* EO. **(A)** Soft agar motility assay; **(B)** Umbrella motility test. CTR, control (*L. monocytogenes* strain grown in the absence of EO).

**Figure 2:**
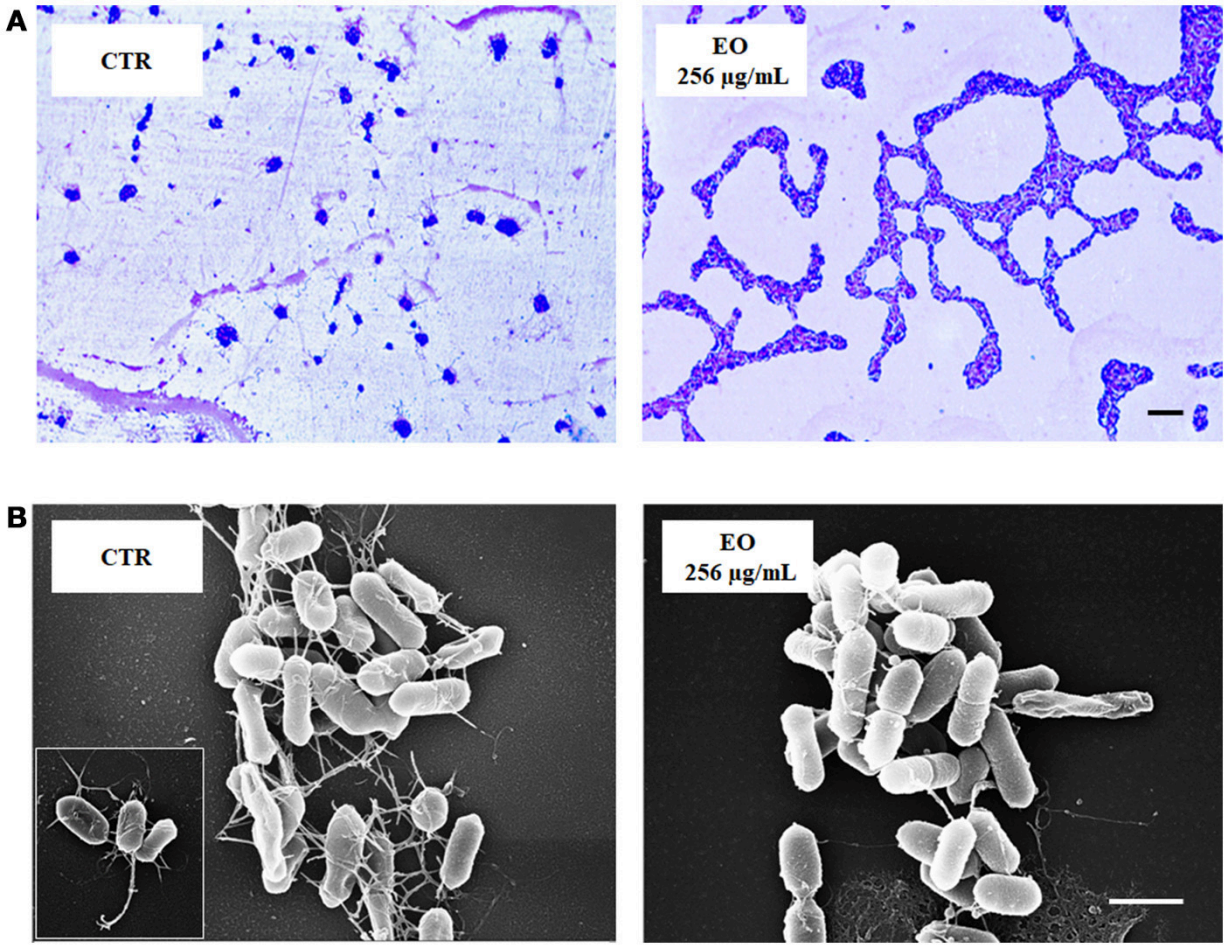
LM and SEM images of *L. monocytogenes* strain #80466 grown in presence of the *C. sativa* EO. **(A)** LM images after staining with Flagella Stain Droppers (magnification: 1000 ×; bar, 5 μm): flagella are stained in pink red; **(B)** SEM images (bar, 1 μm). Insert, “*bouquet*” of listeriae. CTR, control (*L. monocytogenes* strain grown in the absence of EO).

*L. monocytogenes* motility was first evaluated by measuring colony diameter and by the “umbrella motility test” after growth on soft agar containing a sublethal concentration (256 μg/mL) of the *C. sativa* EO (Figures 1A,B). A significant, concentration-dependent, reduction in colony diameter (from 50.0 to 79.8 %) was observed in all strains (Table 4, Figure 1A). Moreover, strains grown in presence of 256 μg/mL of the extract did not show the typical umbrella-like growth (Figure 1B). LM examination after flagella staining showed that, unlike control strains, the listeriae incubated with 256 μg/mL of the *C. sativa* EO formed aggregates with the flagella trapped inside the cluster (Figure 2A). SEM observation confirmed these findings and demonstrated that there were fewer flagella in the extract-incubated strains (Figure 2B).

**Table 4:** Motility of *L. monocytogenes* grown in presence of the *C. sativa* EO.

**Strain #**	**Zone of motility (mm)**		**% reduction**
	**CTR**	**EO (256 μg/mL)**	
60551	18.0 ± 0.6	5.0 ± 0.0*	72.2
66785	21.0 ± 5.2	4.7 ± 0.6*	77.6
75227	9.7 ± 0.6	4.3 ± 0.6*	55.7
97885	10.0 ± 1.0	5.0 ± 0.0*	50.0
82468	23.3 ± 1.2	6.7 ± 0.6*	71.2
02470	22.3 ± 3.8	7.3 ± 0.6*	67.3
09707	23.0 ± 5.3	5.0 ± 0.0*	78.3
56053	21.3 ± 6.7	5.3 ± 0.6*	75.1
31829	14.0 ± 1.0	5.0 ± 1.0*	64.3
77660	23.3 ±6.7	4.7 ± 0.6*	79.8
80466	14.0 ± 1.7	5.0 ± 0.0*	64.3

*Values are mean of two independent experiments. Asterisks indicate significant values compared with control (p ≤ 0.05). CTR, control (L. monocytogenes strains grown in the absence of EO)*.

### Downregulation of *prfA, flaA, motA*, and *motB* in *L. monocytogenes* exposed to the *C. sativa* EO

**Figure 3:**
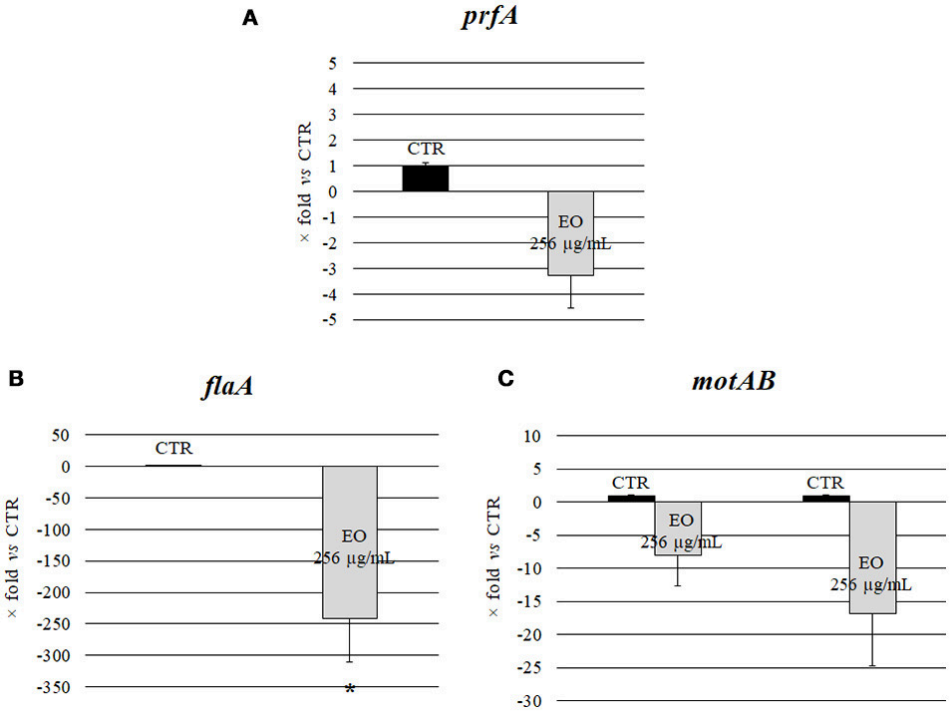
Expression of motility genes of *L. monocytogenes* strain #80466 grown in presence of the *C. sativa* EO. Gene expression was normalized to the expression of the 16S rRNA gene in the same conditions compared to the control and is presented as x-fold: **(A)** *prfA*; **(B)** *flaA*; **(C)** *motA* and *motB*. Mean values and standard deviations of three experiments. Asterisks indicate significant values compared with control (*p* ≤ 0.05). CTR, control (*L. monocytogenes* strain grown in the absence of EO).

Investigation of the relative expression levels of *prfA, flaA, motA* and *motB* genes, after growth in the presence of *C. sativa* EO at 256 μg/mL, by a real-time RT-PCR assay, demonstrated a 3.3, 8.1, and 16.8 reduction in the expression levels of *prfA, motA* and *motB*, respectively, (*p* = 0.0541, *p* = 0.0819, and *p* = 0.0569, respectively). Interestingly, the relative expression of *flaA* also demonstrated a significant 241.5 reduction (*p* = 0.0002) (Figure 3).

### Effects of the *C. sativa* EO on *L. monocytogenes* biofilm formation

**Figure 4:**
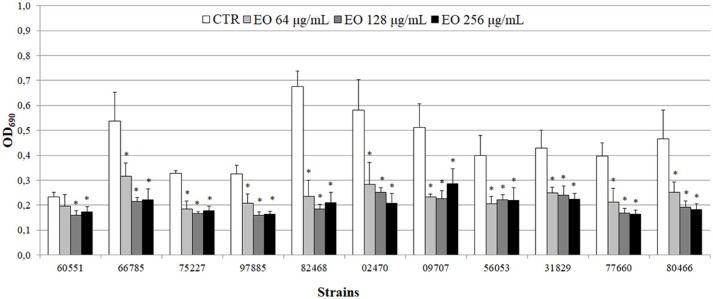
Biofilm production by *L. monocytogenes* in presence of the *C. sativa* EO. Exposure to the three EO concentrations resulted in a shift to a lower producer type in all but one (#60551) of the 11 strains. Values are mean OD_690_ ± SD of three experiments. Asterisks indicate significant values (*p* ≤ 0.05) compared with control (*L. monocytogenes* strain grown in the absence of EO).

All 11 *L. monocytogenes* strains were found to be biofilm producers: 4 strains (#66785, #02470, #09707, and #82468) were strong producers, 6 (#56053, #31829, #77660, #97885, #75227, and #80466) were moderate producers, and one (#60551) was a weak producer. To evaluate the ability of the EO to interfere with biofilm formation, all strains were tested for biofilm production in presence of sublethal (256, 128, and 64 μg/mL) EO concentrations. A reduction in biofilm production was induced by all concentrations in all strains. The 256 μg/mL concentration reduced biofilm production by 25–69%; a similar reduction was observed with 128 μg/mL (from 32 to 73%) and 64 μg/mL (from 15 to 65%) (Figure 4). Except #60551 (weak producer), all the moderate/strong biofilm producers became weak producers at all EO concentrations.

### Effects of exposure of *L. monocytogenes* strains to the *C. sativa* EO on Caco-2 cell invasion efficiency and CPE

**Figure 5:**
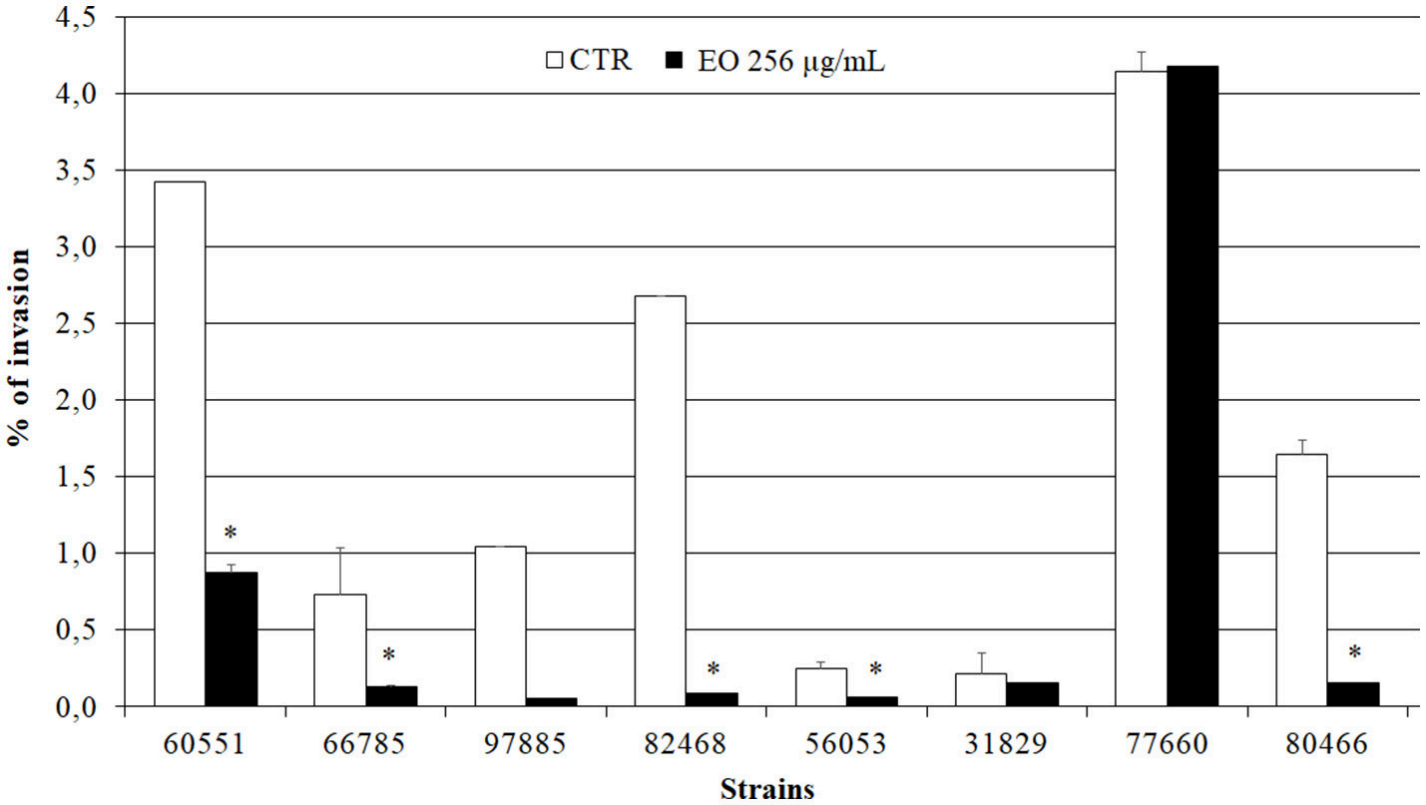
Caco-2 cell invasion by *L. monocytogenes* strains grown in presence of the *C. sativa* EO. Data are expressed as percentage (compared with the initial inoculum) of viable bacteria recovered after 2 h incubation with gentamicin (gentamicin survival test). Each column is the mean of three experiments. Asterisks indicate significant values (*p* ≤ 0.05) compared with control (*L. monocytogenes* strains grown in the absence of EO).

Preliminary Caco-2 infection experiments were conducted to ascertain the ability of *L. monocytogenes* strains to enter Caco-2 cells in the gentamicin survival test. Three strains were excluded from further experiments due to low invasion efficiency; the other 8 strains, which showed an invasion efficiency up to 4.1% of the initial inoculum, were used in infection inhibition experiments. Remarkably, the invasion efficiency of Caco-2 cells by 7/8 strains was strongly reduced (from 25.9 to 97%) when bacteria were grown in presence of 256 μg/mL of the *C. sativa* EO (Figure 5).

**Figure 6:**
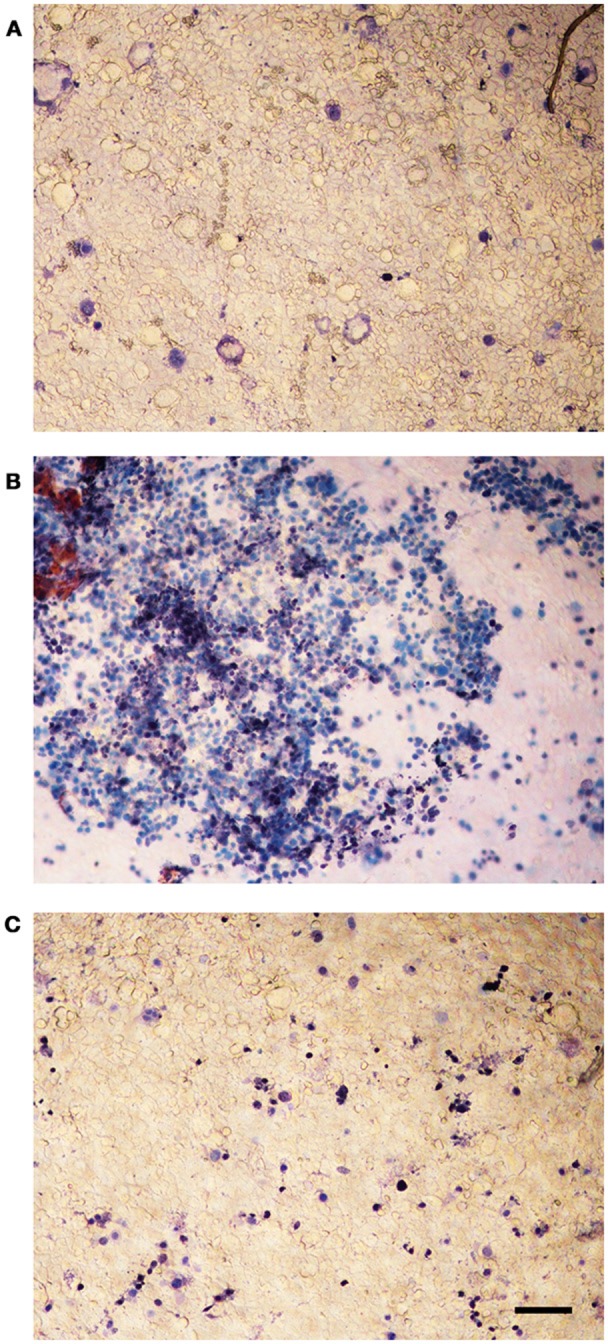
Trypan blue assay on Caco-2 monolayers infected with *L. monocytogenes* grown in presence of the *C. sativa* EO. **(A)** uninfected control; **(B)** monolayers infected with *L. monocytogenes* #80466 not incubated with the EO; **(C)** monolayers infected with *L. monocytogenes* #80466 grown in presence of 256 μg/mL of the EO (magnification: 100 ×; bar, 400 μm).

The viability of infected Caco-2 cells was assessed with trypan blue, which stains dead cells but is actively excluded from viable eukaryotic cells. The test demonstrated a dramatic CPE reduction in *L. monocytogenes* strains grown in presence of 256 μg/mL of the *C. sativa* EO (Figure 6), in particular, the monolayers infected with the extract-exposed strains were actually indistinguishable from the uninfected monolayers, whereas the CPE of control monolayers, i.e., those infected with strains grown without the EO, took the form of large, almost contiguous, stained areas.

### Survival of *G. mellonella* larvae infected with *L. monocytogenes* grown in presence of the *C. sativa* EO

**Figure 7:**
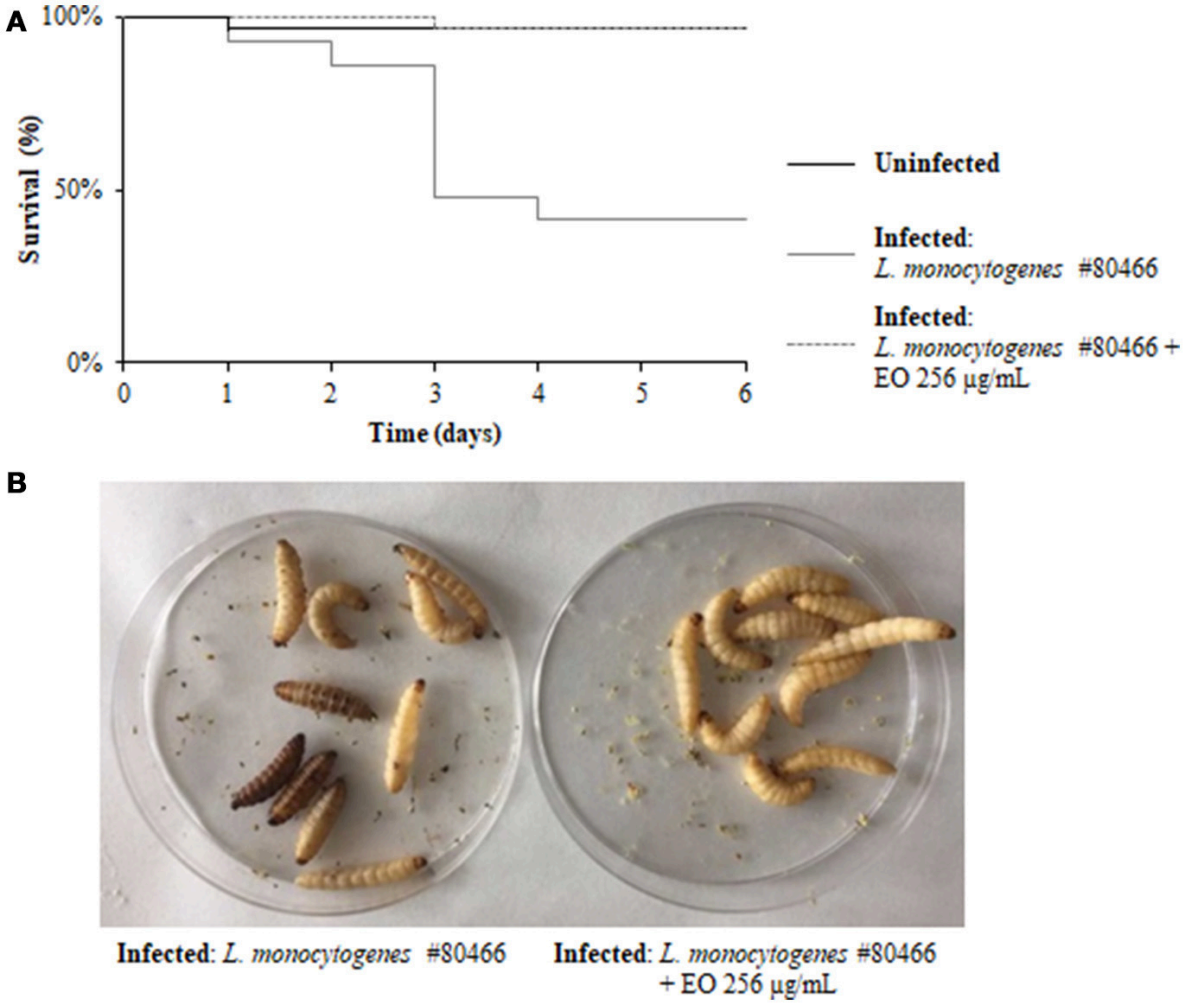
Survival of *G. mellonella* larvae infected with *L. monocytogenes* strain #80466 grown in presence of the *C. sativa* EO. **(A)** Kaplan-Meier survival curves of *G. mellonella* larvae after 6 days from injection with *L. monocytogenes* strain #80466 are shown. No more than one control larvae injected with PBS died in any given trial (data not shown); **(B)** Dead (black and motionless) and viable (yellowish and moving) larvae.

The ability of a sublethal concentration of the *C. sativa* EO to attenuate *L. monocytogenes* virulence was tested *in vivo* using the *G. mellonella* model. Larvae were infected with *L. monocytogenes* strains grown with and without (control) 256 μg/mL of the *C. sativa* EO. Larvae were considered dead when they turned black and did not respond to touch. On day 6 after infection with listeriae, larval mortality was about 50% in the group infected with the strains grown without the EO whereas, remarkably, survival rates greater than 90% (*p* = 0.0009) were seen in the group infected with the strains grown with the EO (Figures 7A,B). PBS (negative control) did not result in larval mortality (data not shown).

## Discussion

*C. sativa* L. has been a source of food, fuel, paper, and building materials, a textile fiber, and a folk medicine remedy for thousands of years, and was eventually abandoned due to its high content in psychotropic compounds. Currently, industrial varieties containing low Δ9-THC concentrations are being studied for their powerful bioactive phytochemicals (Andre et al., 2016).

The antibacterial activity of freshly extracted EO from industrial *C. sativa* varieties has been assessed by Nissen et al. (2010) against Gram-positive and Gram-negative bacteria, mostly food-borne pathogens. In this study, GC-MS analysis of the EO obtained by distillation of the inflorescences and leaves of the variety Futura 75 disclosed a chemical composition similar to the one reported by Nissen et al. (2010) for this variety. Susceptibility experiments, carried out by the microdilution method, demonstrated that the EO and its two main components, α-pinene and myrcene, showed moderate bactericidal activity against clinical strains of *L. monocytogenes*.

The study also evaluated the effects of sublethal concentrations of the *C. sativa* EO on *L. monocytogenes* virulence traits such as motility, biofilm production, and cell invasion. Motility was assessed by phenotypic (umbrella and soft agar motility assay) and molecular (real time RT-PCR) approaches and by LM and SEM observation. Both motility tests demonstrated that, after growth in presence of the *C. sativa* EO, all listerial strains became non-motile. Remarkably, flagella staining of treated cells showed aggregates of listeriae with the flagella trapped inside the aggregates; SEM demonstrated that the flagella were adherent to the cell rather than free, as in control specimens. Real time RT-PCR experiments showed a downregulation of flagellar motility genes *flaA* (encoding flagellin) and *motA* and *motB* (encoding a part of the flagellar motor). A reduced expression levels of *prfA*, the transcriptional activator of genes involved in cell invasion, intracellular surviving, and spreading to neighboring cells, was also demonstrated after growth in the presence of *C. sativa* EO. Inhibition of *L. monocytogenes* motility by natural products has also been reported with trans-cinnamaldehyde from *Cinnamomum zeylandicum*, carvacrol, and thymol, the main components of oregano and thyme EO (Upadhyay et al., 2012). In the Gram-negative pathogens *Salmonella* Typhimurium and *Escherichia coli* O157:H7, carvacrol affects motility through loss of flagellum functionality (Inamuco et al., 2012) and inhibition of flagellin synthesis (Burt et al., 2007), respectively.

Since flagella and flagellum-mediated motility are important virulence traits of *L. monocytogenes* for initial surface attachment and subsequent biofilm formation (Lemon et al., 2007) as well as for cell invasion (Dons et al., 2004), we tested the hypothesis that a reduction of motility by sublethal concentrations of the *C. sativa* EO would affect biofilm-forming and cell invasion ability. The hypothesis was successfully demonstrated. Remarkably, the number of intracellular listeriae (gentamicin survival test) was almost zero when Caco-2 cells were infected with listeriae exposed to the EO and their viability was unaffected (trypan blue test). The present results therefore clearly demonstrate that sublethal concentrations of the *C. sativa* EO can affect the virulence of *L. monocytogenes in vitro*.

*In vivo* experiments using *G. mellonella* larvae as a model (Rakic Martinez et al., 2017) were performed to establish whether incubation of listeriae with sublethal concentrations of the *C. sativa* EO would prevent larval infection. The test has recently emerged as a promising model to assess the virulence of numerous human pathogens, including *L. monocytogenes*. In our study, larvae infected with *L. monocytogenes* grown in presence of sublethal EO concentrations showed a significantly higher survival compared with larvae infected with untreated listeriae. Thus, the *in vivo* experiments confirmed the protective activity of the *C. sativa* EO against *L. monocytogenes* infection.

Since LLO production is critical to *L. monocytogenes* pathogenesis in *G. mellonella* (Joyce and Gahan, 2010) we could hypothesize that the protective activity of the *C. sativa* EO is due to the attenuation of this virulence factor.

Targeting microbial virulence rather than survival is an exciting novel strategy with the potential to reduce the evolutionary pressure for the development of resistance. Food contamination by *L. monocytogenes* remains a major concern for the food processing industry, particularly the plants making ready-to-eat and processed food. The present work provides a baseline in the study of the anti-virulence properties of the EO of *C. sativa* against *L. monocytogenes*. Further studies are needed to understand if it could be used as an alternative agent for the control of *L. monocytogenes* in food processing plants.

## Author contributions

BF, GM, and EM conceived the study. BF, GM, EM, AP, and MR designed the experiments. GM, EM, AP, and AG performed the experiments. BF, GM, EM, AP, GF, TB, MR, and AG analyzed the data. BF, GM, and EM wrote the paper. All authors approved the manuscript.

### Conflict of interest statement

The authors declare that the research was conducted in the absence of any commercial or financial relationships that could be construed as a potential conflict of interest.
